# Bis(2-{[bis­(dimethyl­amino)­methyl­idene]amino-κ*N*}benzene­sulfonato-κ*N*)copper(II)

**DOI:** 10.1107/S1600536812046387

**Published:** 2012-11-14

**Authors:** Adam Neuba, Ulrich Flörke, Gerald Henkel

**Affiliations:** a Universität Paderborn, Fakultät für Naturwissenschaften, Department Chemie, Warburger Strasse 100, 33098 Paderborn, Germany

## Abstract

The mol­ecular structure of the title compound, [Cu(C_11_H_16_N_3_O_3_S)_2_], shows the Cu^II^ atom with a distorted square-planar coordination geometry from the N_2_O_2_ donor set of the two chelating 2-{[bis­(dimethyl­amino)­methyl­idene]amino}­benzene­sulfonate ligands. The Cu^II^ atom lies 0.065 (1) Å above the N_2_O_2_ plane and the Cu—O [2 × 1.945 (2) Å] and Cu—N bond lengths [1.968 (3) and 1.962 (3) Å] lie in expected ranges. The two aromatic ring planes make a dihedral angle of 85.48 (1)°.

## Related literature

For bifunctional peralkyl­ated guanidine ligands, see: Bienemann *et al.* (2011 ▶); Börner *et al.* (2009 ▶); Herres-Pawlis *et al.* (2005 ▶, 2009 ▶); Neuba *et al.* (2008 ▶, 2010 ▶); Pohl *et al.* (2000 ▶); Raab *et al.* (2003 ▶); Wittmann *et al.* (2001 ▶). For guanidine–sulfur hybrids to mimic the structural and physical as well as functional characteristics of the Cu^II^ atom in cytochrome c oxidase and N_2_O reductase, see: Neuba *et al.* (2011 ▶, 2012 ▶). For related structures with Cu(N_2_O_2_) motifs, see: Robinson *et al.* (2004 ▶).
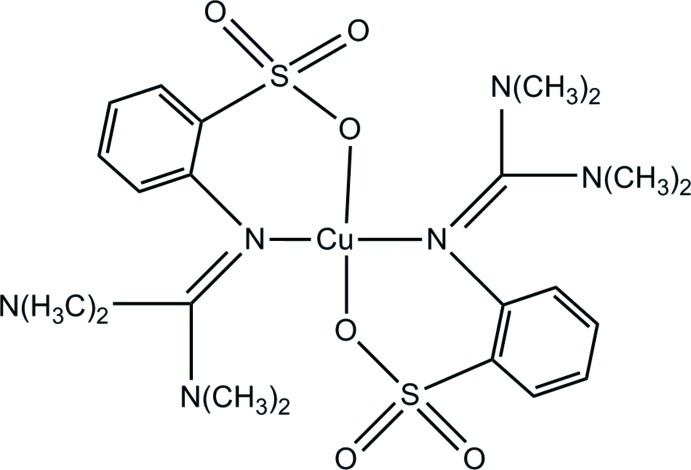


## Experimental

### 

#### Crystal data

[Cu(C_11_H_16_N_3_O_3_S)_2_]
*M* *_r_* = 604.20
Orthorhombic, 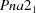

*a* = 19.940 (3) Å
*b* = 12.2947 (14) Å
*c* = 10.9508 (14) Å
*V* = 2684.7 (6) Å^3^
*Z* = 4
Mo *K*α radiation
μ = 1.02 mm^−1^
*T* = 120 K
0.29 × 0.23 × 0.20 mm

#### Data collection

Bruker SMART APEX diffractometer
Absorption correction: multi-scan (*SADABS*; Sheldrick, 2004 ▶) *T*_min_ = 0.757, *T*_max_ = 0.822
22901 measured reflections
6315 independent reflections
4939 reflections with *I* > 2σ(*I*)
*R*_int_ = 0.063

#### Refinement

*R*[*F* ^2^ > 2σ(*F* ^2^)] = 0.044
*wR*(*F* ^2^) = 0.093
*S* = 1.02
6315 reflections
342 parameters
1 restraint
H-atom parameters constrained
Δρ_max_ = 0.63 e Å^−3^
Δρ_min_ = −0.29 e Å^−3^
Absolute structure: Flack (1983 ▶), 2953 Friedel pairs
Flack parameter: 0.021 (12)

### 

Data collection: *SMART* (Bruker, 2002 ▶); cell refinement: *SAINT* (Bruker, 2002 ▶); data reduction: *SAINT*; program(s) used to solve structure: *SHELXTL* (Sheldrick, 2008 ▶); program(s) used to refine structure: *SHELXTL*; molecular graphics: *SHELXTL*; software used to prepare material for publication: *SHELXTL* and local programs.

## Supplementary Material

Crystal structure: contains datablock(s) I, global. DOI: 10.1107/S1600536812046387/aa2076sup1.cif

Structure factors: contains datablock(s) I. DOI: 10.1107/S1600536812046387/aa2076Isup2.hkl

Additional supplementary materials:  crystallographic information; 3D view; checkCIF report

## Figures and Tables

**Fig. 1.**
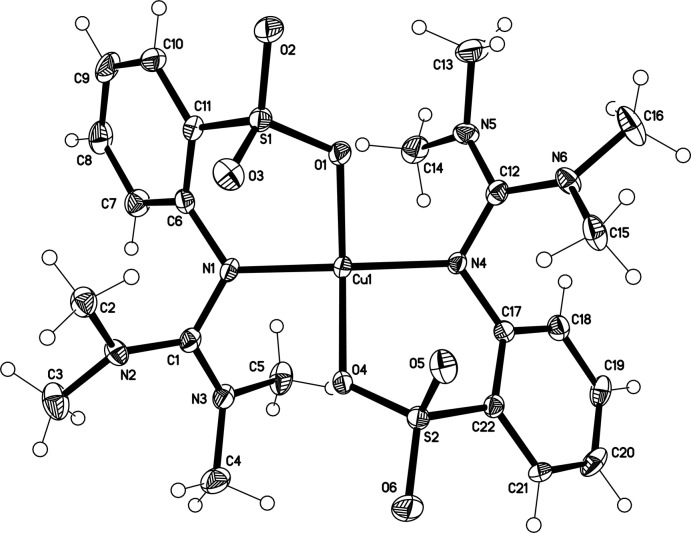
Molecular structure of the title compound. Anistropic displacement parameters are shown at the 50% probability level.

## supplementary crystallographic information

### Experimental

In a first step the mixed-valent copper thiolate complex [Cu_6_(NGuaS)_6_](PF_6_)_2_ [NGuaS = 2-(1,1,3,3-tetramethylguanidino)benzenethiolate, C_11_H_16_N_3_S] was synthesized (Neuba *et al.*, 2011): reaction of 1,1,3,3-tetramethyl-2-(2-(tritylthio)phenyl)guanidine (510 mg, 1.1 mmol) with [Cu(MeCN)_4_]PF_6_ (186.2 mg, 0.5 mmol) dissolved in 5 ml of *ABS*. MeCN led to a deep blue/green solution. The reaction mixture was stirred for a period of 30 min. at room temperature followed by heating under reflux for 30 min. After cooling the solution was filtered. Second step: slow diffusion of air at -20°C to the filtrate leads after several weeks to dark red crystals of [Cu(C_11_H_16_N_3_O_3_S)_2_] suitable for X-ray diffraction. We suppose a copper mediated oxidation of the *o*-tetramethylguanidinobenzenethiolate ligand to the corresponding benzenesulfonate.

### Refinement

H atoms were clearly identified in difference syntheses, refined at idealized positions riding on the carbon atoms with isotropic displacement parameters *U*_iso_(H) = 1.2U_eq_(C) or 1.5U_eq_(methyl C). All CH_3_ hydrogen atoms were allowed to rotate but not to tip.

### Crystal data

**Table:**

[Cu(C_11_H_16_N_3_O_3_S)_2_]	*F*(000) = 1260
*M**_r_* = 604.20	*D*_x_ = 1.495 Mg m^−^^3^
Orthorhombic, *P**n**a*2_1_	Mo *K*α radiation, λ = 0.71073 Å
Hall symbol: P 2c -2n	Cell parameters from 1013 reflections
*a* = 19.940 (3) Å	θ = 2.5–23.8°
*b* = 12.2947 (14) Å	µ = 1.02 mm^−^^1^
*c* = 10.9508 (14) Å	*T* = 120 K
*V* = 2684.7 (6) Å^3^	Block, red
*Z* = 4	0.29 × 0.23 × 0.20 mm

### Data collection

**Table:**

Bruker SMART APEX diffractometer	6315 independent reflections
Radiation source: sealed tube	4939 reflections with *I* > 2σ(*I*)
Graphite monochromator	*R*_int_ = 0.063
φ and ω scans	θ_max_ = 27.9°, θ_min_ = 2.0°
Absorption correction: multi-scan (*SADABS*; Sheldrick, 2004)	*h* = −26→25
*T*_min_ = 0.757, *T*_max_ = 0.822	*k* = −16→14
22901 measured reflections	*l* = −14→14

### Refinement

**Table:**

Refinement on *F*^2^	Secondary atom site location: difference Fourier map
Least-squares matrix: full	Hydrogen site location: difference Fourier map
*R*[*F*^2^ > 2σ(*F*^2^)] = 0.044	H-atom parameters constrained
*wR*(*F*^2^) = 0.093	*w* = 1/[σ^2^(*F*_o_^2^) + (0.0383*P*)^2^] where *P* = (*F*_o_^2^ + 2*F*_c_^2^)/3
*S* = 1.02	(Δ/σ)_max_ < 0.001
6315 reflections	Δρ_max_ = 0.63 e Å^−^^3^
342 parameters	Δρ_min_ = −0.29 e Å^−^^3^
1 restraint	Absolute structure: Flack (1983), 2953 Friedel pairs
Primary atom site location: structure-invariant direct methods	Flack parameter: 0.021 (12)

### Special details

**Table:**

Geometry. All s.u.'s (except the s.u. in the dihedral angle between two l.s. planes) are estimated using the full covariance matrix. The cell s.u.'s are taken into account individually in the estimation of s.u.'s in distances, angles and torsion angles; correlations between s.u.'s in cell parameters are only used when they are defined by crystal symmetry. An approximate (isotropic) treatment of cell s.u.'s is used for estimating s.u.'s involving l.s. planes.
Refinement. Refinement of *F*^2^ against ALL reflections. The weighted *R*-factor *wR* and goodness of fit *S* are based on *F*^2^, conventional *R*-factors *R* are based on *F*, with *F* set to zero for negative *F*^2^. The threshold expression of *F*^2^ > σ(*F*^2^) is used only for calculating *R*-factors(gt) *etc*. and is not relevant to the choice of reflections for refinement. *R*-factors based on *F*^2^ are statistically about twice as large as those based on *F*, and *R*- factors based on ALL data will be even larger.

### Fractional atomic coordinates and isotropic or equivalent isotropic displacement parameters (Å^2^)

**Table:**

	*x*	*y*	*z*	*U*_iso_*/*U*_eq_	
Cu1	0.375129 (18)	0.12574 (3)	0.29666 (4)	0.01655 (10)	
S1	0.25801 (4)	0.02454 (7)	0.41807 (8)	0.01986 (19)	
S2	0.49473 (4)	0.22745 (7)	0.40833 (8)	0.01868 (18)	
O1	0.28279 (12)	0.12436 (19)	0.3539 (2)	0.0220 (6)	
O2	0.18969 (13)	0.0382 (2)	0.4554 (3)	0.0313 (7)	
O3	0.30433 (13)	−0.0077 (2)	0.5122 (2)	0.0267 (6)	
O4	0.46904 (11)	0.12775 (18)	0.3447 (2)	0.0211 (6)	
O5	0.45077 (14)	0.2585 (2)	0.5066 (2)	0.0254 (6)	
O6	0.56389 (12)	0.2136 (2)	0.4400 (3)	0.0321 (7)	
N1	0.37774 (14)	−0.0306 (2)	0.2583 (2)	0.0173 (6)	
N2	0.44573 (14)	−0.1771 (2)	0.3195 (3)	0.0219 (7)	
N3	0.48764 (15)	−0.0454 (2)	0.1876 (3)	0.0235 (7)	
N4	0.37182 (14)	0.2806 (2)	0.2535 (3)	0.0165 (6)	
N5	0.26055 (14)	0.2943 (3)	0.1932 (3)	0.0226 (7)	
N6	0.30479 (14)	0.4264 (2)	0.3212 (3)	0.0230 (7)	
C1	0.43704 (18)	−0.0852 (3)	0.2543 (3)	0.0195 (8)	
C2	0.40296 (18)	−0.2039 (3)	0.4219 (4)	0.0270 (8)	
H2A	0.3828	−0.1373	0.4545	0.040*	
H2B	0.4297	−0.2392	0.4858	0.040*	
H2C	0.3674	−0.2535	0.3949	0.040*	
C3	0.4881 (2)	−0.2670 (3)	0.2783 (4)	0.0370 (10)	
H3A	0.5061	−0.2504	0.1972	0.055*	
H3B	0.4613	−0.3338	0.2742	0.055*	
H3C	0.5252	−0.2772	0.3359	0.055*	
C4	0.55762 (19)	−0.0533 (3)	0.2252 (4)	0.0357 (11)	
H4A	0.5607	−0.0962	0.3004	0.054*	
H4B	0.5755	0.0198	0.2397	0.054*	
H4C	0.5837	−0.0888	0.1606	0.054*	
C5	0.4752 (2)	0.0283 (3)	0.0868 (4)	0.0321 (10)	
H5A	0.4288	0.0199	0.0589	0.048*	
H5B	0.5059	0.0115	0.0195	0.048*	
H5C	0.4824	0.1034	0.1138	0.048*	
C6	0.31920 (17)	−0.0947 (3)	0.2379 (3)	0.0196 (8)	
C7	0.3188 (2)	−0.1734 (3)	0.1472 (4)	0.0278 (9)	
H7A	0.3586	−0.1886	0.1028	0.033*	
C8	0.2598 (2)	−0.2305 (3)	0.1211 (4)	0.0330 (10)	
H8A	0.2597	−0.2837	0.0582	0.040*	
C9	0.2016 (2)	−0.2100 (3)	0.1860 (4)	0.0335 (10)	
H9A	0.1618	−0.2493	0.1676	0.040*	
C10	0.20118 (18)	−0.1333 (3)	0.2766 (4)	0.0265 (9)	
H10A	0.1613	−0.1195	0.3214	0.032*	
C11	0.25995 (15)	−0.0753 (3)	0.3028 (4)	0.0198 (7)	
C12	0.31265 (18)	0.3351 (3)	0.2543 (3)	0.0204 (8)	
C13	0.19238 (19)	0.3016 (4)	0.2378 (5)	0.0399 (12)	
H13A	0.1925	0.3323	0.3204	0.060*	
H13B	0.1724	0.2288	0.2397	0.060*	
H13C	0.1661	0.3486	0.1835	0.060*	
C14	0.2704 (2)	0.2234 (4)	0.0906 (4)	0.0324 (10)	
H14A	0.3151	0.2359	0.0558	0.049*	
H14B	0.2362	0.2383	0.0286	0.049*	
H14C	0.2668	0.1475	0.1174	0.049*	
C15	0.34810 (19)	0.4524 (3)	0.4234 (4)	0.0292 (9)	
H15A	0.3693	0.3858	0.4536	0.044*	
H15B	0.3215	0.4854	0.4889	0.044*	
H15C	0.3828	0.5037	0.3968	0.044*	
C16	0.2584 (2)	0.5139 (3)	0.2842 (4)	0.0388 (11)	
H16A	0.2398	0.4973	0.2035	0.058*	
H16B	0.2827	0.5831	0.2809	0.058*	
H16C	0.2218	0.5193	0.3438	0.058*	
C17	0.42974 (17)	0.3458 (3)	0.2312 (3)	0.0166 (7)	
C18	0.42834 (19)	0.4263 (3)	0.1423 (3)	0.0231 (8)	
H18A	0.3881	0.4391	0.0981	0.028*	
C19	0.4846 (2)	0.4882 (3)	0.1173 (4)	0.0279 (10)	
H19A	0.4830	0.5418	0.0549	0.033*	
C20	0.5430 (2)	0.4726 (3)	0.1823 (4)	0.0300 (9)	
H20A	0.5810	0.5169	0.1664	0.036*	
C21	0.54617 (16)	0.3927 (3)	0.2703 (3)	0.0223 (9)	
H21A	0.5866	0.3811	0.3142	0.027*	
C22	0.49011 (15)	0.3290 (2)	0.2949 (4)	0.0178 (6)	

### Atomic displacement parameters (Å^2^)

**Table:**

	*U*^11^	*U*^22^	*U*^33^	*U*^12^	*U*^13^	*U*^23^
Cu1	0.01765 (18)	0.01225 (17)	0.0197 (2)	−0.00136 (15)	0.0002 (2)	−0.0011 (2)
S1	0.0212 (4)	0.0173 (4)	0.0211 (5)	−0.0007 (3)	0.0063 (4)	−0.0007 (4)
S2	0.0192 (4)	0.0167 (4)	0.0201 (4)	0.0001 (3)	−0.0057 (4)	0.0007 (4)
O1	0.0205 (13)	0.0171 (13)	0.0285 (14)	−0.0015 (10)	0.0076 (11)	−0.0011 (11)
O2	0.0256 (14)	0.0276 (15)	0.0408 (18)	−0.0018 (12)	0.0135 (12)	0.0006 (13)
O3	0.0312 (15)	0.0294 (15)	0.0196 (15)	0.0041 (11)	0.0017 (12)	−0.0027 (11)
O4	0.0197 (12)	0.0135 (12)	0.0302 (15)	0.0007 (10)	−0.0074 (10)	0.0005 (10)
O5	0.0356 (16)	0.0233 (14)	0.0172 (14)	0.0032 (12)	−0.0013 (12)	0.0026 (11)
O6	0.0263 (14)	0.0326 (15)	0.0375 (18)	−0.0030 (12)	−0.0152 (13)	0.0035 (14)
N1	0.0208 (15)	0.0131 (14)	0.0179 (16)	−0.0041 (12)	0.0043 (11)	−0.0022 (11)
N2	0.0244 (15)	0.0207 (16)	0.0207 (18)	0.0054 (12)	0.0022 (13)	−0.0001 (13)
N3	0.0235 (16)	0.0179 (16)	0.0291 (18)	−0.0016 (13)	0.0101 (14)	−0.0048 (14)
N4	0.0173 (14)	0.0135 (14)	0.0186 (15)	−0.0018 (13)	−0.0016 (11)	0.0028 (11)
N5	0.0164 (15)	0.0264 (18)	0.0248 (18)	0.0016 (13)	−0.0050 (13)	0.0017 (14)
N6	0.0247 (15)	0.0230 (16)	0.0212 (19)	0.0083 (13)	−0.0048 (13)	−0.0012 (13)
C1	0.0224 (18)	0.0156 (17)	0.0204 (19)	−0.0027 (15)	0.0026 (14)	−0.0069 (14)
C2	0.0304 (19)	0.025 (2)	0.026 (2)	0.0015 (16)	0.0050 (18)	0.0047 (18)
C3	0.050 (3)	0.027 (2)	0.034 (3)	0.0107 (18)	0.015 (2)	0.002 (2)
C4	0.023 (2)	0.032 (2)	0.052 (3)	−0.0043 (18)	0.0107 (19)	−0.009 (2)
C5	0.045 (3)	0.023 (2)	0.029 (2)	−0.004 (2)	0.0171 (19)	0.0026 (19)
C6	0.0225 (18)	0.0146 (17)	0.0217 (19)	0.0022 (14)	−0.0016 (15)	−0.0006 (15)
C7	0.031 (2)	0.025 (2)	0.028 (2)	−0.0015 (17)	0.0000 (17)	−0.0042 (17)
C8	0.045 (3)	0.0205 (19)	0.033 (2)	−0.004 (2)	−0.008 (2)	−0.0078 (19)
C9	0.034 (2)	0.023 (2)	0.044 (3)	−0.0131 (18)	−0.010 (2)	−0.001 (2)
C10	0.0195 (17)	0.0264 (19)	0.034 (3)	−0.0017 (15)	−0.0020 (16)	0.0059 (18)
C11	0.0229 (16)	0.0155 (15)	0.0210 (18)	−0.0027 (13)	0.0007 (18)	0.0012 (17)
C12	0.0229 (19)	0.0182 (18)	0.0200 (19)	−0.0030 (15)	−0.0038 (14)	0.0055 (14)
C13	0.019 (2)	0.042 (3)	0.058 (3)	−0.004 (2)	−0.0020 (19)	0.010 (2)
C14	0.030 (2)	0.031 (2)	0.036 (3)	−0.005 (2)	−0.0153 (18)	0.004 (2)
C15	0.041 (2)	0.0206 (19)	0.026 (2)	0.0099 (16)	−0.0083 (19)	−0.0069 (18)
C16	0.054 (3)	0.030 (2)	0.032 (2)	0.0243 (19)	−0.010 (2)	−0.004 (2)
C17	0.0178 (17)	0.0164 (17)	0.0156 (18)	0.0010 (14)	−0.0017 (14)	−0.0009 (14)
C18	0.0263 (19)	0.0196 (19)	0.023 (2)	0.0016 (16)	−0.0028 (16)	0.0005 (15)
C19	0.040 (2)	0.0171 (19)	0.027 (2)	−0.0073 (17)	0.0046 (18)	0.0057 (16)
C20	0.030 (2)	0.028 (2)	0.032 (2)	−0.0157 (18)	0.0079 (18)	−0.0024 (18)
C21	0.0128 (15)	0.0208 (18)	0.033 (3)	−0.0030 (14)	0.0020 (14)	−0.0012 (16)
C22	0.0192 (15)	0.0154 (15)	0.0190 (16)	−0.0009 (12)	0.0014 (17)	−0.0023 (17)

### Geometric parameters (Å, º)

**Table:**

Cu1—O4	1.945 (2)	C4—H4C	0.9800
Cu1—O1	1.945 (2)	C5—H5A	0.9800
Cu1—N4	1.962 (3)	C5—H5B	0.9800
Cu1—N1	1.968 (3)	C5—H5C	0.9800
S1—O2	1.432 (3)	C6—C7	1.386 (5)
S1—O3	1.440 (3)	C6—C11	1.400 (5)
S1—O1	1.498 (2)	C7—C8	1.399 (6)
S1—C11	1.761 (4)	C7—H7A	0.9500
S2—O6	1.432 (3)	C8—C9	1.385 (6)
S2—O5	1.439 (3)	C8—H8A	0.9500
S2—O4	1.500 (2)	C9—C10	1.369 (6)
S2—C22	1.764 (4)	C9—H9A	0.9500
N1—C1	1.361 (4)	C10—C11	1.402 (5)
N1—C6	1.426 (4)	C10—H10A	0.9500
N2—C1	1.348 (4)	C13—H13A	0.9800
N2—C2	1.447 (5)	C13—H13B	0.9800
N2—C3	1.463 (4)	C13—H13C	0.9800
N3—C1	1.338 (4)	C14—H14A	0.9800
N3—C5	1.450 (5)	C14—H14B	0.9800
N3—C4	1.458 (5)	C14—H14C	0.9800
N4—C12	1.357 (4)	C15—H15A	0.9800
N4—C17	1.427 (4)	C15—H15B	0.9800
N5—C12	1.333 (5)	C15—H15C	0.9800
N5—C14	1.436 (5)	C16—H16A	0.9800
N5—C13	1.447 (5)	C16—H16B	0.9800
N6—C12	1.350 (4)	C16—H16C	0.9800
N6—C15	1.449 (5)	C17—C18	1.389 (5)
N6—C16	1.475 (4)	C17—C22	1.407 (5)
C2—H2A	0.9800	C18—C19	1.383 (5)
C2—H2B	0.9800	C18—H18A	0.9500
C2—H2C	0.9800	C19—C20	1.377 (6)
C3—H3A	0.9800	C19—H19A	0.9500
C3—H3B	0.9800	C20—C21	1.378 (5)
C3—H3C	0.9800	C20—H20A	0.9500
C4—H4A	0.9800	C21—C22	1.391 (4)
C4—H4B	0.9800	C21—H21A	0.9500
			
O4—Cu1—O1	145.52 (11)	H5B—C5—H5C	109.5
O4—Cu1—N4	94.88 (10)	C7—C6—C11	118.6 (3)
O1—Cu1—N4	93.10 (11)	C7—C6—N1	120.2 (3)
O4—Cu1—N1	92.56 (11)	C11—C6—N1	121.1 (3)
O1—Cu1—N1	94.89 (11)	C6—C7—C8	120.1 (4)
N4—Cu1—N1	153.75 (11)	C6—C7—H7A	120.0
O2—S1—O3	115.99 (17)	C8—C7—H7A	120.0
O2—S1—O1	110.59 (15)	C9—C8—C7	120.6 (4)
O3—S1—O1	110.46 (15)	C9—C8—H8A	119.7
O2—S1—C11	107.89 (16)	C7—C8—H8A	119.7
O3—S1—C11	107.88 (16)	C10—C9—C8	120.1 (4)
O1—S1—C11	103.18 (15)	C10—C9—H9A	119.9
O6—S2—O5	115.89 (17)	C8—C9—H9A	119.9
O6—S2—O4	110.14 (15)	C9—C10—C11	119.6 (4)
O5—S2—O4	110.87 (15)	C9—C10—H10A	120.2
O6—S2—C22	107.76 (15)	C11—C10—H10A	120.2
O5—S2—C22	107.88 (15)	C6—C11—C10	121.0 (3)
O4—S2—C22	103.47 (15)	C6—C11—S1	120.1 (3)
S1—O1—Cu1	118.07 (14)	C10—C11—S1	118.9 (3)
S2—O4—Cu1	117.69 (14)	N5—C12—N6	119.6 (3)
C1—N1—C6	115.7 (3)	N5—C12—N4	119.3 (3)
C1—N1—Cu1	120.8 (2)	N6—C12—N4	121.0 (3)
C6—N1—Cu1	123.5 (2)	N5—C13—H13A	109.5
C1—N2—C2	121.7 (3)	N5—C13—H13B	109.5
C1—N2—C3	123.0 (3)	H13A—C13—H13B	109.5
C2—N2—C3	114.0 (3)	N5—C13—H13C	109.5
C1—N3—C5	121.0 (3)	H13A—C13—H13C	109.5
C1—N3—C4	122.9 (3)	H13B—C13—H13C	109.5
C5—N3—C4	114.9 (3)	N5—C14—H14A	109.5
C12—N4—C17	115.3 (3)	N5—C14—H14B	109.5
C12—N4—Cu1	120.5 (2)	H14A—C14—H14B	109.5
C17—N4—Cu1	124.0 (2)	N5—C14—H14C	109.5
C12—N5—C14	120.9 (3)	H14A—C14—H14C	109.5
C12—N5—C13	122.6 (4)	H14B—C14—H14C	109.5
C14—N5—C13	115.5 (3)	N6—C15—H15A	109.5
C12—N6—C15	122.2 (3)	N6—C15—H15B	109.5
C12—N6—C16	122.0 (3)	H15A—C15—H15B	109.5
C15—N6—C16	115.1 (3)	N6—C15—H15C	109.5
N3—C1—N2	119.9 (3)	H15A—C15—H15C	109.5
N3—C1—N1	119.5 (3)	H15B—C15—H15C	109.5
N2—C1—N1	120.5 (3)	N6—C16—H16A	109.5
N2—C2—H2A	109.5	N6—C16—H16B	109.5
N2—C2—H2B	109.5	H16A—C16—H16B	109.5
H2A—C2—H2B	109.5	N6—C16—H16C	109.5
N2—C2—H2C	109.5	H16A—C16—H16C	109.5
H2A—C2—H2C	109.5	H16B—C16—H16C	109.5
H2B—C2—H2C	109.5	C18—C17—C22	118.0 (3)
N2—C3—H3A	109.5	C18—C17—N4	120.3 (3)
N2—C3—H3B	109.5	C22—C17—N4	121.7 (3)
H3A—C3—H3B	109.5	C19—C18—C17	121.0 (4)
N2—C3—H3C	109.5	C19—C18—H18A	119.5
H3A—C3—H3C	109.5	C17—C18—H18A	119.5
H3B—C3—H3C	109.5	C20—C19—C18	120.4 (4)
N3—C4—H4A	109.5	C20—C19—H19A	119.8
N3—C4—H4B	109.5	C18—C19—H19A	119.8
H4A—C4—H4B	109.5	C19—C20—C21	120.0 (3)
N3—C4—H4C	109.5	C19—C20—H20A	120.0
H4A—C4—H4C	109.5	C21—C20—H20A	120.0
H4B—C4—H4C	109.5	C20—C21—C22	120.0 (3)
N3—C5—H5A	109.5	C20—C21—H21A	120.0
N3—C5—H5B	109.5	C22—C21—H21A	120.0
H5A—C5—H5B	109.5	C21—C22—C17	120.6 (3)
N3—C5—H5C	109.5	C21—C22—S2	119.5 (3)
H5A—C5—H5C	109.5	C17—C22—S2	119.9 (2)
			
O2—S1—O1—Cu1	179.11 (16)	C7—C6—C11—C10	−0.5 (6)
O3—S1—O1—Cu1	49.3 (2)	N1—C6—C11—C10	175.5 (3)
C11—S1—O1—Cu1	−65.74 (18)	C7—C6—C11—S1	−179.6 (3)
O4—Cu1—O1—S1	−68.1 (3)	N1—C6—C11—S1	−3.5 (5)
N4—Cu1—O1—S1	−171.33 (17)	C9—C10—C11—C6	−0.1 (6)
N1—Cu1—O1—S1	33.69 (18)	C9—C10—C11—S1	179.0 (3)
O6—S2—O4—Cu1	178.28 (16)	O2—S1—C11—C6	171.3 (3)
O5—S2—O4—Cu1	48.7 (2)	O3—S1—C11—C6	−62.6 (3)
C22—S2—O4—Cu1	−66.76 (18)	O1—S1—C11—C6	54.3 (3)
O1—Cu1—O4—S2	−66.2 (2)	O2—S1—C11—C10	−7.7 (4)
N4—Cu1—O4—S2	36.56 (18)	O3—S1—C11—C10	118.3 (3)
N1—Cu1—O4—S2	−168.63 (17)	O1—S1—C11—C10	−124.8 (3)
O4—Cu1—N1—C1	−10.2 (3)	C14—N5—C12—N6	158.1 (3)
O1—Cu1—N1—C1	−156.5 (3)	C13—N5—C12—N6	−33.5 (5)
N4—Cu1—N1—C1	96.2 (4)	C14—N5—C12—N4	−25.5 (5)
O4—Cu1—N1—C6	167.3 (3)	C13—N5—C12—N4	142.9 (4)
O1—Cu1—N1—C6	21.0 (3)	C15—N6—C12—N5	155.9 (3)
N4—Cu1—N1—C6	−86.3 (4)	C16—N6—C12—N5	−33.5 (5)
O4—Cu1—N4—C12	−156.2 (3)	C15—N6—C12—N4	−20.5 (5)
O1—Cu1—N4—C12	−9.8 (3)	C16—N6—C12—N4	150.2 (4)
N1—Cu1—N4—C12	97.8 (3)	C17—N4—C12—N5	133.9 (3)
O4—Cu1—N4—C17	17.6 (3)	Cu1—N4—C12—N5	−51.8 (4)
O1—Cu1—N4—C17	164.0 (3)	C17—N4—C12—N6	−49.8 (4)
N1—Cu1—N4—C17	−88.3 (4)	Cu1—N4—C12—N6	124.6 (3)
C5—N3—C1—N2	158.8 (3)	C12—N4—C17—C18	−41.9 (4)
C4—N3—C1—N2	−34.6 (5)	Cu1—N4—C17—C18	144.0 (3)
C5—N3—C1—N1	−23.0 (5)	C12—N4—C17—C22	140.0 (3)
C4—N3—C1—N1	143.6 (3)	Cu1—N4—C17—C22	−34.1 (4)
C2—N2—C1—N3	159.1 (3)	C22—C17—C18—C19	−0.1 (5)
C3—N2—C1—N3	−34.5 (5)	N4—C17—C18—C19	−178.2 (3)
C2—N2—C1—N1	−19.1 (5)	C17—C18—C19—C20	−1.4 (6)
C3—N2—C1—N1	147.3 (4)	C18—C19—C20—C21	2.0 (6)
C6—N1—C1—N3	131.5 (3)	C19—C20—C21—C22	−1.0 (6)
Cu1—N1—C1—N3	−50.8 (4)	C20—C21—C22—C17	−0.5 (5)
C6—N1—C1—N2	−50.3 (4)	C20—C21—C22—S2	−179.7 (3)
Cu1—N1—C1—N2	127.4 (3)	C18—C17—C22—C21	1.0 (5)
C1—N1—C6—C7	−41.8 (5)	N4—C17—C22—C21	179.1 (3)
Cu1—N1—C6—C7	140.6 (3)	C18—C17—C22—S2	−179.8 (3)
C1—N1—C6—C11	142.3 (3)	N4—C17—C22—S2	−1.7 (5)
Cu1—N1—C6—C11	−35.4 (4)	O6—S2—C22—C21	−12.1 (3)
C11—C6—C7—C8	0.9 (6)	O5—S2—C22—C21	113.7 (3)
N1—C6—C7—C8	−175.1 (4)	O4—S2—C22—C21	−128.8 (3)
C6—C7—C8—C9	−0.7 (6)	O6—S2—C22—C17	168.7 (3)
C7—C8—C9—C10	0.1 (7)	O5—S2—C22—C17	−65.5 (3)
C8—C9—C10—C11	0.3 (6)	O4—S2—C22—C17	52.1 (3)
